# Anthophilous insects' seasonal variation in Corsican thermo-Mediterranean shrubland maquis

**DOI:** 10.3897/BDJ.13.e144560

**Published:** 2025-03-07

**Authors:** Pierre-Yves Maestracci, Laurent Plume, Marc Gibernau

**Affiliations:** 1 ENGIE-Lab-CRIGEN, Stains, France ENGIE-Lab-CRIGEN Stains France; 2 CNRS – University of Corsica - Laboratory Sciences for the Environment (UMR 6134 SPE), Natural Resources Project, Ajaccio, France CNRS – University of Corsica - Laboratory Sciences for the Environment (UMR 6134 SPE), Natural Resources Project Ajaccio France; 3 University Paris-Panthéon-Assas, Laboratory Management Research (Largepa), Paris, France University Paris-Panthéon-Assas, Laboratory Management Research (Largepa) Paris France

**Keywords:** Floral visitors, seasonality, Coleoptera, Hymenoptera, Diptera, Lepidoptera, Corsica

## Abstract

**Background:**

In any ecosystems, seasonality is a key factor conditioning the temporal variation on an annual scale in combination with differences in the organism phenology. This seasonality is marked in the Mediterranean Region with four contrasting seasons: a hot, dry summer, a mild winter and sometimes a very rainy spring and autumn. With a large surface area and its large range of habitats from seaside to alpine biotopes, Corsica Island represents a biodiversity hotspot with a high rate of endemism. Amongst diverse groups, insects, notably the main orders of pollinators composed of Coleoptera, Hymenoptera, Diptera and Lepidoptera, represent a large proportion of the insular richness.

**New information:**

Our sampling effort focused on the insects from these four orders visiting flowers in a characteristic thermo-Mediterranean vegetation. Our database is an insight into the Corsican anthophilous insects biodiversity from three sites separated by a few kilometres in the region of Ajaccio (SW Corsica) during nine consecutive months in 2023, completing our database for the year 2022 published in this journal. In total, 3714 specimens were sampled in 2023 and 311 species or morpho-species identified from 154 genera and 50 families. Coleoptera were by far the most abundant order representing about 54% of the sampled specimens. The most diverse order was the Hymenoptera representing 44% of the species. Our continuous survey has shown that these orders vary between seasons both in terms of abundance and diversity, resulting in changing communities.

## Introduction

Insects pollinators and flowering plants communities represent major ecosystems components with respectively 350,000 and 400,000 known species, mainly (98.4%) belonging to the four orders Lepidoptera, Coleoptera, Hymenoptera and Diptera (Ollerton 2017). Their interactions contribute to the ecosytem pollination function and are primordial both for conservation biology and evolution of many terrestrial ecosystems (Layek et al. 2022), these links being amongst the most important to the evolution of life on Earth (Wardhaugh 2015). Seasonality and variations in habitats compositions contribute to the dynamism of anthophilous insect communities (Olesen et al. 2008, Dupont et al. 2009, Poisot et al. 2015). Taking the time factor into account allows us to understand the functioning of associated ecosystems more precisely and to go beyond a simplified static vision (CaraDonna et al. 2021).

Southern Europe with its varied habitats, their structure and the seasonal weather is home to a significant richness of animal and plant species, particularly in the Mediterranean Region which represents a biodiversity hotspot (Mittermeier 2004, Ollerton 2017). This climate classified as Csa (Hot-summer Mediterranean climate in the Koppen climate classification system) is made up of four contrasting seasons: a hot and dry summer, a mild winter and a (sometimes very) rainy spring and autumn. In recent years in Corsica Island, significant studies on insect diversity have been carried out including the MNHN “Planète revisitée” expeditions (Ichter 2021, Ichter 2022, Touroult et al. 2023) and OCIC (Observatoire Conservatoire des Invertébrés de Corse) works (Jiroux 2019, Cornuel-Willermoz and Andrei-Ruiz 2021, Cornuel-Willermoz and Lebard 2024, Le Divelec et al. 2024).

However, there is still little work focusing on the pollination function (Mengual et al. 2023, Maestracci et al. 2024). By focusing on insect-plant interactions and capturing insects on sight by sweep-net those visiting wildflowers along transects and static observations, our study is more in line with an ecosystem approach rather than an exhaustive inventory of entomofauna.

This paper aims to: (1) make public the data of anthophilous insects sampled in a thermo-Mediterranean schrubland maquis over 9 months and (2) show the seasonal dynamic of anthophilous insects corteges throughout the 2023 year.

## General description

### Purpose

Our aim is to publish in open access the records of anthophilous insects collected during a 9-months study on plant-pollinator interactions in Corsica in 2023. This dataset completes our first 2022 inventory publihsed in this journal (Maestracci et al. 2024).

## Project description

### Title

Anthophilous insects' seasonal variation in Corsican thermo-Mediterranean shrubland maquis in 2023.

### Personnel

Pierre-Yves Maestracci; Laurent Plume and Marc Gibernau.

### Study area description

Sampling was conducted on three sites near Ajaccio namely Loretto, Suartello and Vignola (Table 1) representing the ecological compensation zones for Loregaz, an industrial project that has impacted three protected species (*Testudohermanni* Gmelin, 1789; *Serapiasneglecta* De Not., 1844; *Serapiasparviflora* Parl., 1837). These sites are managed by an association, the Conservatoire d'Espaces Naturels de Corse, on behalf of ENGIE, the company behind the project. On each site, the main vegetation is the Mediterranean maquis and the sampling design took into account the environmental differences within and amongst sites in order to have a good representation of the vegetation.

### Design description

The data published in this paper are part of a larger research project including plant-pollinator insect interaction networks (Nicolson and Wright 2017) and their dynamics over time (Burkle and Alarcón 2011).

### Funding

UMR SPE 6134, CPER project N°40137 “BiodivCorse – Explorer la biodiversité de la Corse” (Collectivité de Corse – Ministère de la Cohésion du territoire et des Relations avec les Collectivités territoriales), ENGIE Solutions/Storengy and CIFRE doctoral programme (ENGIE/Lab. CRIGEN-Univ. Corsica-Univ. Panthéon-Assas).

## Sampling methods

### Study extent

The surveys were carried out on three sites on 2.5 ha effective sampling area out of a total of 22 ha of compensation sites (Table 1).

### Sampling description

On each of the three sites every two weeks from mid-February to mid-November 2023, all anthophilous insects were collected on sight by sweep net during the different time slots of the day : Morning (9 h-12 h), mid-day (12 h-14 h) and afternoon (14 h-17 h). The samples were taken on favourable days, without rain and when the wind did not exceed 30 km/h. For each time slot, two pollinating insect sampling methods were carried out consecutively at the three study sites (Loretto, Suartello and Vignola) to target not only their diversity, but also their interaction, which is not possible with the usual pan-traps method (Westphal et al. 2008). The first method was dynamic and all the insects visiting flowers were collected along two transects (30 m long and 2 m wide) for 30 min/transect. The transects crossed the different types of vegetation in the studied area. The second method was static and consisted in capturing all the insects visiting the flowers for a period of 5 minutes on two different plants of the same species. For each field session, six different characteristic flowering species were selected depending on their abundance in the environment, resulting in a total of 12 flowers observed during a total period of 1 hour. The selected six species changed throughout the year according to their flowering seasons. The same protocol was used between 2022 and 2023, whose data were published in a previous publication; for more details on the protocol, please refer to it (Maestracci et al. 2024). In 2023, 168 transects (equivalent to 84 hours) were sampled with the dynamic method and 78 flowering plants observations were achieved using the static method (equivalent to 78 hours).

### Quality control

Morphological identifications (Hymenoptera and Lepidoptera: P-Y. Maestracci and A. Cornuel-Willermoz, Diptera and Coleoptera: L. Plume, Syrphidae: V. Sarthou and T. Lebard) and several CO1 barcoding (unpubl. data).

Morphological identifications were possible thanks to taxonomical reference works, insect checklists and Corsican studies:

- Hymenoptera (Benoist 1929, Benoist 1931, Mauss and Treiber 1994, Amiet et al. 1999, Amiet et al. 2001, Amiet et al. 2004, Amiet et al. 2007, Amiet et al. 2010, Patiny and Terzo 2010, Terzo et al. 2013, Pauly 2015, Michez et al. 2019, Rasmont et al. 2021) with checklist (Ghisbain et al. 2023, Reverté et al. 2023) and local study (Le Divelec et al. 2024);
- Diptera, especially Syrphidae (on French fauna: Seguy 1961, Speight 2020, Speight and Langlois 2020, Speight and Lebard 2020, Speight et al. 2021, Sarthou et al. 2021; on Western Europe hoverflies: Van Veen 2004 and Mediterranean species: Grković et al. 2016, Van Steenis et al. 2017, Malidžan et al. 2022) with checklists (Dirickx 1994, Reverté et al. 2023) and local studies (Goeldlin de Tiefenau and Lucas 1981, Mengual et al. 2023, Cornuel-Willermoz and Lebard 2024, Plume et al. 2024);
- Coleoptera (Albouy and Richard 2017, Jiroux 2019) and checklists (Tronquet 2014);
- and diurnal Lepidoptera (Cooper et al. 2022) and checklists (Wiemers et al. 2018).

Lists by the National Museum of Natural History of Paris from la “Planète revisitée” Corsican missions were also consulted (Ichter 2021, Ichter 2022, Touroult et al. 2023) or by the Observatoire Conservatoire des Invertébrés de Corse (OCIC) (Cornuel-Willermoz and Andrei-Ruiz 2021).

## Geographic coverage

### Description

**Figure 1.**
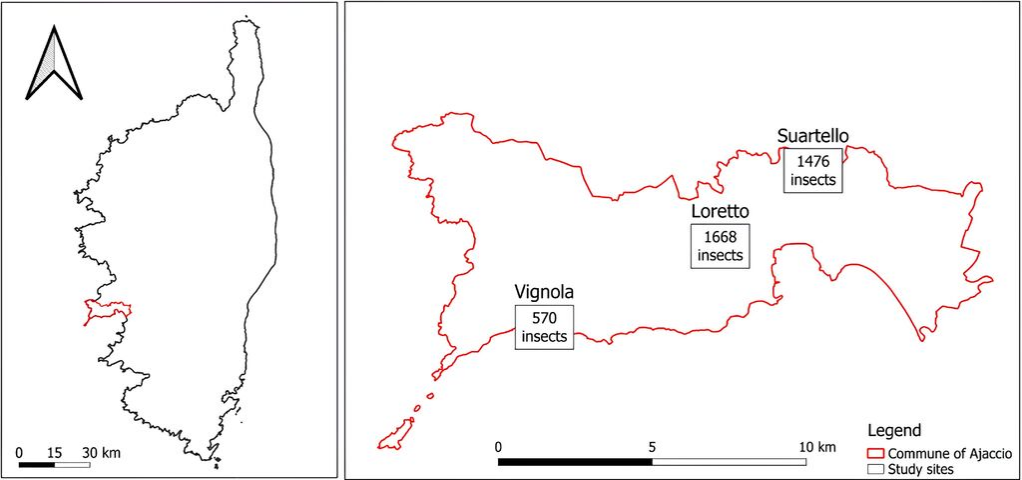
Geographical localisation of the three studied sites and total specimen abundances sampled per site.

**Figure 2.**
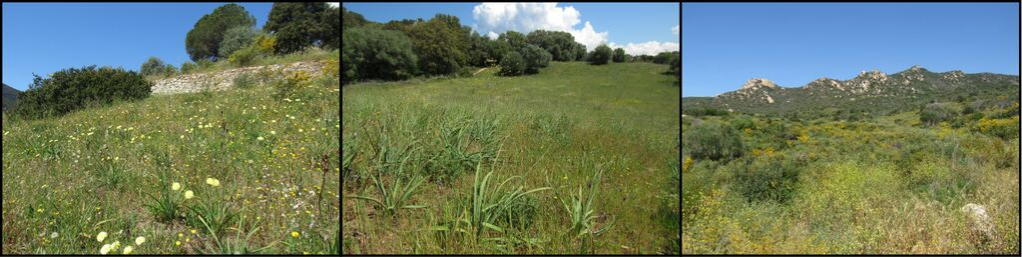
Floral habitats of the three sites on April (Loretto on the left, Suartello in the middle and Vignola on the right).

South-west Corsica, Ajaccio Region. The Loretto site, located a few hundred metres from the city centre of Ajaccio adjoining the industrial Loregaz site, is made up of a plant mosaic, alternating open areas and groves. The Suartello site, located on the edge of a wooded area, is made up of an open environment (e.g. grassland) and a plant mosaic environment. The Vignola site facing the sea (ca. 200 m inland) was partly degraded by heavy rotary grinding in 2018, 4 years before the study. The proximity of the sites to each other makes it possible to consider their average temperatures and precipitation as being similar. Thus, they have a warm temperate climate with an average annual temperature of 16.3°C. However, some differences exist; Vignola is more exposed to sea spray and Suartello is slightly shadier due to the presence of trees on one side (Figs 1, 2).

## Taxonomic coverage

### Description

In 2023, 3714 insects were collected with 311 morphospecies that could be identified as part of 50 different families (including one as "not identified" category grouping together unidentified Coleoptera, Diptera and Hymenoptera specimens) amongst the four main orders of anthophilous insects : Coleoptera [2027 specimens; 69 morpho-species], Hymenoptera [1125; 138], Diptera [450; 83] and Lepidoptera [109; 19]. Few specimens belonging to Hemiptera [3; 1] order were also collected.

### Taxa included

**Table:** 

Rank	Scientific Name	Common Name
kingdom	Animalia	Animals
phylum	Arthropoda	Arthropods
class	Insecta	Insects
order	Coleoptera	Beetles
order	Diptera	Flies
order	Hymenoptera	Bees and wasps
order	Lepidoptera	Butterflies
family	Andrenidae	Mining bees
family	Anthomyiidae	Root-maggot flies
family	Apidae	Bees
family	Bombyliidae	Bee flies
family	Braconidae	Braconid wasps
family	Brentidae	Straight-snouted weevils
family	Buprestidae	Jewel beetles
family	Calliphoridae	Blow flies
family	Cerambycidae	Longhorn beetles
family	Chalcidoidae	Chalcid wasps
family	Chrysididae	Cuckoo emerald wasps
family	Chrysomelidae	Leaf beetles
family	Coccinellidae	Ladybirds
family	Colletidae	Plasterer bees
family	Conopidae	Thick-headed flies
family	Crabronidae	Square-headed wasps
family	Curculionidae	Snout beetles
family	Dermestidae	Skin beetles
family	Empididae	Dagger flies
family	Ephydridae	Shore flies
family	Halictidae	Sweat bees
family	Heleomyzidae	Sun flies
family	Hesperidae	Skippers
family	Ichneumonidae	Ichneumon wasps
family	Leucospidae	Leucospid wasps
family	Lycaenidae	Gossamer-winged butterflies
family	Megachilidae	Leafcutter bees
family	Meloidae	Blister beetles
family	Melyridae	Soft-winged flower beetles
family	Mordellidae	Tumbling flower beetles
family	Muscidae	House flies
family	Nitidulidae	Sap beetles
family	Nymphalidae	Brush-footed butterflies
family	Oedemeridae	False blister beetles
family	Papilionidae	Swallowtail butterflies
family	Philanthidae	Philanth wasps
family	Pieridae	Butterflies
family	Pompilidae	Spider wasps
family	Rhiniidae	Nose flies
family	Scarabaeidae	Scarab beetles
family	Scoliidae	Scoliid wasps
family	Sphecidae	Digger wasps
family	Sphingidae	Sphinx moths
family	Stratiomyidae	Soldier flies
family	Syrphidae	Hoverflies
family	Tachinidae	Tachinid flies
family	Tenthredinidae	Common sawflies
family	Tiphiidae	Tiphiid flower wasps
family	Vespidae	Wasps

## Temporal coverage

**Data range:** 2023-2-10 – 2023-11-14.

### Notes

Specimens were collected every two weeks from February to November 2023.

## Collection data

### Collection name

SPE_Insects_Collection

### Collection identifier

Laurent Plume & Pierre-Yves Maestracci

### Specimen preservation method

Dried and pinned specimens and specimens in 70° alcohol.

## Usage licence

### Usage licence

Creative Commons Public Domain Waiver (CC-Zero)

## Data resources

### Data package title

Anthophilous insects of thermo-Mediterranean shrubland maquis (Ajaccio, Corsica, France)

### Resource link

https://doi.org/10.5281/zenodo.14499399

### Number of data sets

1

### Data set 1.

#### Data set name

Anthophilous_insects_2023_data_Corsica_France

#### Data format

TXT (linefeed only)

#### Data format version

Darwin core

#### Description

A total of 3714 occurrences of anthophilous insects from Ajaccio Region, south-west Corsica. This dataset includes authors own identifications with geo-localisations.

**Data set 1.** 

Column label	Column description
occurrenceID	Unique code of data occurrence.
basisOfRecord	The specific nature of the data record (i.e. PreservedSpecimen or HumanObservation).
eventDate	Event date in format YYYY-MM-DDTHHMM.
year	Year of capture.
month	Month of capture.
verbatimEventDate	Date of capture, in format YYYY-MM-DD at HHMM.
scientificName	Lowest taxonomic rank possible, usually the species name. If the species is unknown, the genus or family names are given.
kingdom	Kingdom (i.e. Animalia).
phylum	Phylum (i.e. Arthropoda).
class	Class (i.e. Insecta).
order	Order.
family	Family name.
genus	Genus name.
specificEpithet	Species epithet of the scientificName.
infraspecificEpithet	Infra-specific epithet of the scientificName (subspecies).
taxonRank	Taxonomic rank of the most specific name in the scientificName.
identifiedBy	Name of the entomologist who identified the specimen, if indicated by the label.
dateIdentified	Year of identification.
decimalLatitude	Geographic latitude (in decimal degrees) of the location.
decimalLongitude	Geographic longitude (in decimal degrees) of the location.
geodeticDatum	Coordinate system and set of reference points upon which the geographic coordinates are based (i.e. WGS 84).
country	Country of capture (France).
countryCode	Two letter country code of the specimen origin (FR).
locality	Location of capture, usually the locality (three locality: Loretto, Suartello and Vignola).
stateProvince	French departmental administrative division (Corse-Du-Sud).
municipality	French municipality (Ajaccio).
institutionCode	Place where the specimen is held (University of Corsica - CRIGEN-ENGIE).
catalogNumber	Box identifier.
organismQuantity	Number of individuals bearing the same label (usually 1).
organismQuantityType	Individuals.
verbatimIdentification	Species name originally given by the original collector, if different from scientificName.
identificationVerificationStatus	Status of insect verification (1 or 0).
coordinateUncertaintyInMetres	Uncertainty in coordinates (a few hundred metres at most).
georeferencedBy	Identity of the person who added the Latitude and longitude data, usually Maestracci Pierre-Yves.
georeferenceProtocol	How the georeference was computed, i.e. from label data (Locality).
georeferenceSources	Georeference code was inferred from geoportail.fr.
georeferencedDate	Georeference work was performed in 2023.
language	French and English.
collectionCode	Code of the collection (InsectsPollinators).
recordedBy	Name of collector (usually Marc Gibernau).
otherCatalogNumbers	The code use by the institution having ownership of the object(s) or information referred to in the record.

## Additional information

### General discussion

**Anthophilous insect diversity**

In comparison to the sampling of 2022 (Maestracci et al. 2024), the number of collected insects was relatively similar (4012 in 2022 vs. 3714 in 2023) as well as for Coleoptera (2187 in 2022 vs. 2027 in 2023) with only 7.32% of decrease. On the other hand, the numbers of sampled Hymenoptera (1368 in 2022 vs. 1125 in 2023) and Lepidoptera (152 in 2021/22 vs. 109 in 2023) were much lower with a decrease of 17.76% and 28.29%, respectively. On the contrary, many more Diptera were collected (288 in 2022 vs. 450 in 2023) with an increase of 56.25%. In terms of diversity, about 60 news species were identified mainly in the Hymenoptera (+35 species) and the Diptera (+19 species) (Suppl. material 1), consistent with the efforts made to increase species identifications in these orders since the publication of the first database.

The total species richness for each order over the two studied years and the three sampled sites was estimated with iChao-1 using the diversity indices menu of Past software 4.17 (Hammer et al. 2001, Suppl. material 2). Afterwards, our data completeness was calculated for Diptera (66%), Hymenoptera (70%), Coleoptera (80%) and Lepidoptera (98%) (Suppl. material 2). These completeness differences between insect orders are probably due to some bias in our sampling protocol (i.e. active collection by sweep net) for small and/or fast species, as a similar collecting effort was indiscriminately applied to all the orders (i.e. all spotted anthophilous insects were captured). Passive sampling protocols would have given different results as it is a more continuous collecting method, capturing of non-anthophilous invertebrates, but without the floral visit information (Westphal et al. 2008).

The number of anthophilous insects in Corsica is actually not known since no global insect inventory has been conducted and only a few groups have been recently studied/revised. By comparing our data with the well known and recently studied Corsican taxa, our sampling is estimated to cover 42% of the 57 island's diurnal Lepidoptera (Berquier and Andrei-Ruiz 2017) and 37% of wild bees diversity out of a total of 361 reported species (Ropars et al. 2025). We have also 29% of the hoverflies diversity out of a total of 168 species (Cornuel-Willermoz and Lebard 2024) and only 8% of the Buprestidae, Chrysomelidae and Scarabaeidae diversity out of a total of 92, 124 and 66 species, respectively (Jiroux 2019). For the last percentage, it is important to take into account that the three cited Coleoptera families contain many non-anthophilous species that will not be sampled with our method. It also noteworthy that the proportions of ‘rare’ species, represented by singletons in our database, highly vary amongst orders from 4% for Lepidoptera to 39% for Diptera (Suppl. material 2).

**Figure 3.**
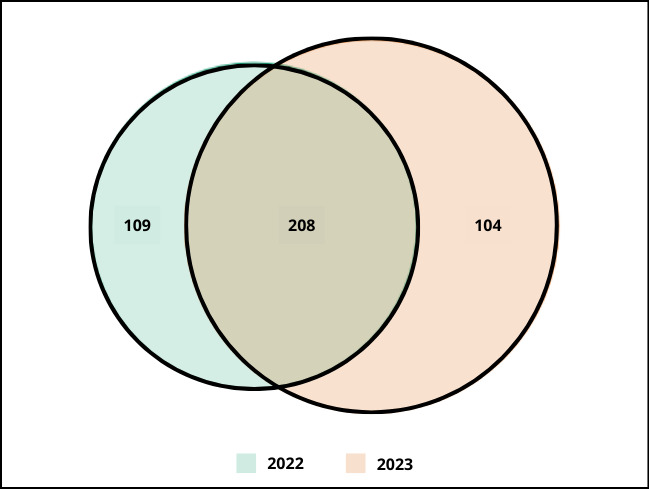
Venn diagram between sampling year of 2022 and 2023 with number of species specific to these two years (to the left and right) and common to both years (in the middle).

It is surprising that our species list is so diverse, considering that less than 3 ha were surveyed over a 9-month period in a limited region representing a mosaic of lowland maquis vegetation. However, only half of the species are common to both years (Fig. 3), with the other half being exclusive to one year and in a balanced manner. This inter-annual variation is well known from various pollinator communities and result from a combination of environmental and ecological factors (Alarcón et al. 2008, Burkle and Irwin 2009, Lázaro et al. 2010, Rohde and Pilliod 2021, Fang et al. 2024). We plan to explore further which drivers affect the studied anthophilous communities in a future paper.

**Figure 4.**
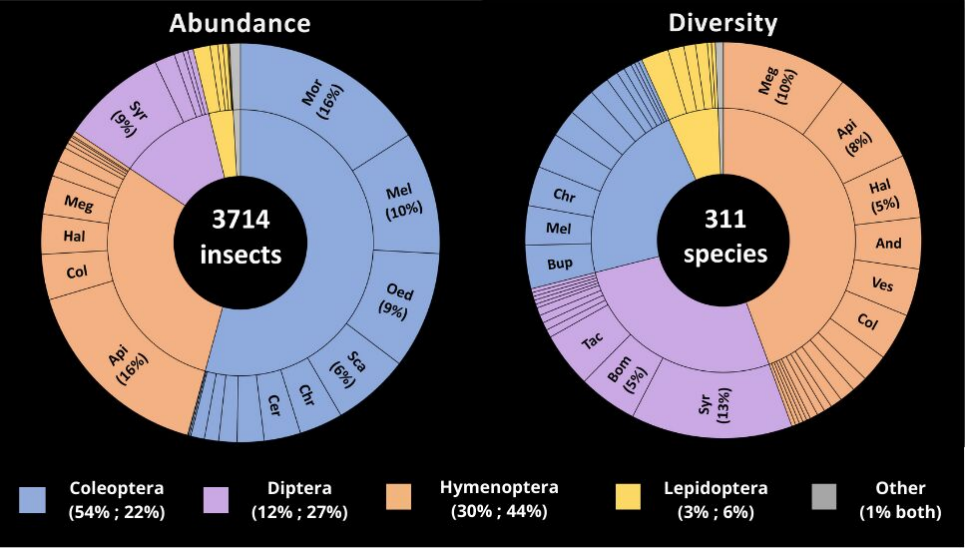
Anthophilous insect families and orders' abundance (on the left) and diversity (on the right) and proportions associated (for orders below, abundance then diversity). For family identities, please refer to Suppl. material 3.

Now analysing our 2023 dataset, in term of abundances, more specimens were collected of Coleoptera (54%) than for any other insect Order (Hymenoptera: 30%, Diptera: 12% and Lepidoptera: 3%) (Fig. 4 - left). The five most numerous families (representing 66% of all the specimens) were the Apidae bees and the Mordellidae beetles (16% each), the Melyridae beetles (10%), the Syrphidae flies and the Oedemeridae beetles (9% each) and the Scarabaeidae beetles (6%) (Fig. 4 - left).

The diversity of the studied anthophilous insect communities was dominated by the order of the Hymenoptera (44% of the morpho-species) followed by the Diptera (27%), the Coleoptera (22%) and, lastly, the Lepidoptera (6%) (Fig. 4 - right). The three richest families were the Syrphidae flies (13%), the Megachilidae bees (10%) and the Apidae bees (8%) (Fig. 4 - right). Hymenoptera, in particular, wild bees, are the most important pollinators in these ecosystems (Kantsa et al. 2023) with a greater specific richness founded in the Mediterranean climatic zones (Michener 2007, Ollerton 2017, Ghisbain et al. 2023). It is estimated that approximately 720 bee species are present in the Mediterranean Basin (Ropars et al. 2020). On the other hand, although Coleoptera are more diverse in the Tropics, they are not negligible in Mediterranean ecosystems (Proctor et al. 1996, Bernhardt 2000, Moore et al. 2018, Li et al. 2021).

This work contributes to fill the existing knowledge gap on anthophilous insects, both for the French Pollinator National Plan and the Corsican Pollinator Territorial Plan (Cornuel-Willermoz and Andrei-Ruiz 2021). Networks analyses will be perfomed on this dataset to detect in the studied ecosystems, amongst other things, the keystone pollinating species, but also the rare sensitive ones (Maestracci et al., unpubl.).

**Seasonal variations of the anthophilous insect communities**

**Figure 5.**
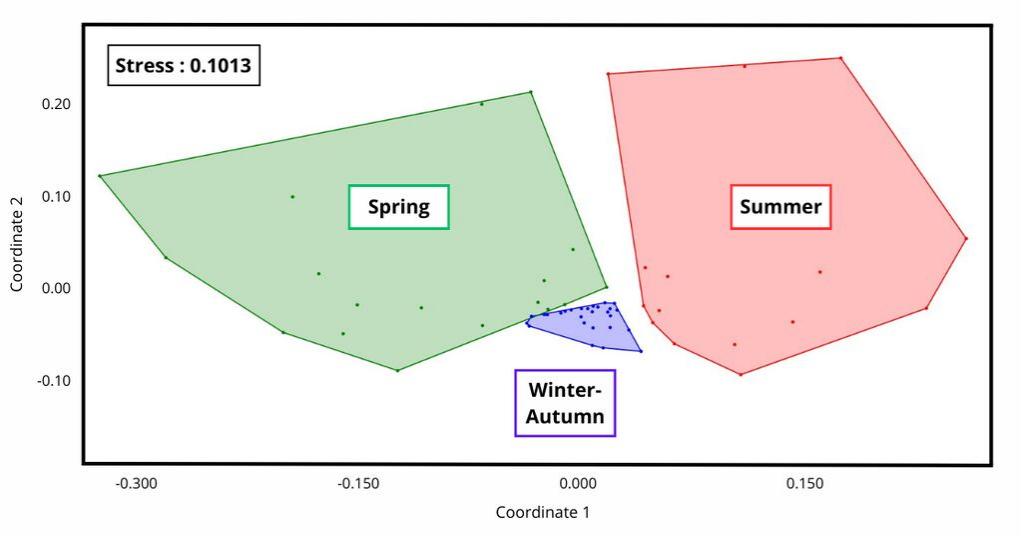
NMDS representation of insect communities on anthophilous insects abundance (euclidian distance on data-transformed ln (abundance+1)) for each field sampling (each point represents data for one date-one site). Colour codes according to the three chosen seasons. Stress associated (means of evaluating the adequacy of our ordination) and ordination coordinates in X and Y.

Another important finding of our study is the seasonality and dynamics of anthophilous species throughout the year. According to preliminary analyses (Maestracci et al., unpubl.), we pooled the bimonthly anthophilous communities into three seasonal groups (winter-autumn, spring and summer). The temporal variation in insect assemblages (ln(abundances+1)) was visualised using a non-metric multidimensional scaling (NMDS) analysis with Euclidean distances (Hammer et al. 2001). The NMDS plot demonstrates clear separation between insect communities amongst the chosen seasons, with minimum overlap (Fig. 5). The species composition of these groups was tested using a non-parametric analysis of similarities (ANOSIM, Suppl. material 4). The species assemblages within these groups were significantly different (ANOSIM test: R² = 0.74, p = 0.0001), with pairwise comparisons showing significant differences after Bonferroni correction (p ≤ 0.013).

**Figure 6.**
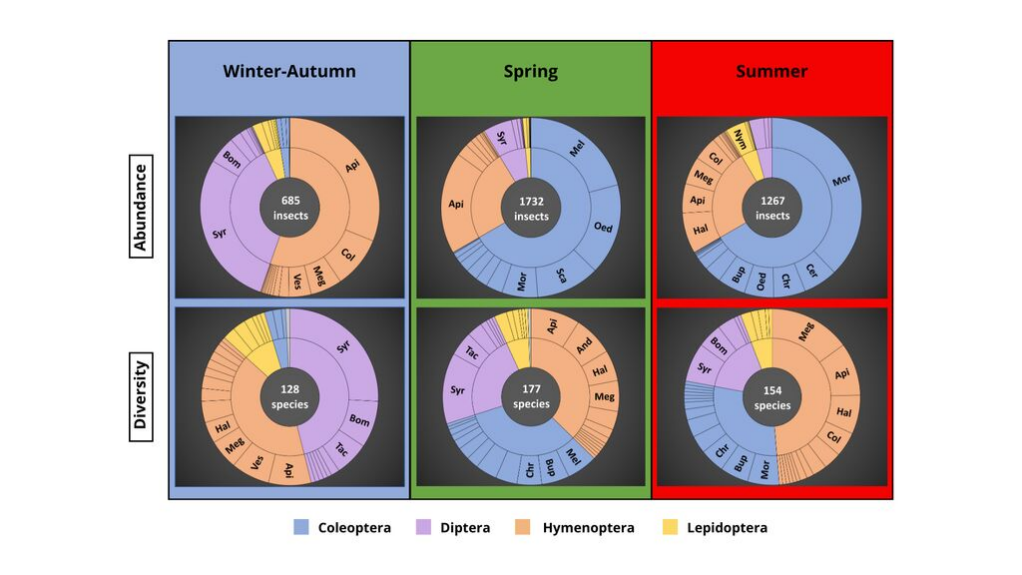
Anthophilous insects' abundance and diversity according to NMDS groups.

Anthophilous insects of the ‘winter-autumn’ community were less numerous with about half the abundances of the two other seasonal communities (Fig. 6 – upper graphs), but its diversity was only slightly less rich (Fig. 6 – lower graphs).

The ‘winter-autumn’ community was characterised by a co-dominance of Hymenoptera, particularly Apidae and Diptera, particularly Syrphidae and Bombyliidae (Fig. 6 – left column). The spring community of anthophilous insects was dominated by the abundance of Coleoptera (~ 67% of the specimens) from the Melyridae, Oedemeridae and Scarabaeidae families; followed by Hymenoptera (Apidae, 18%). The species diversity of the spring community was characterised by Hymenoptera and Coleoptera (respectively ~ 37% and ~ 33%) followed by Diptera species (~ 23%) (Fig. 6 – centre column). The summer community of anthophilous insects was also dominated by the abundance of Coleoptera (~ 67% of the specimens), but from different families (Mordellidae, Cerambycidae, Chrysomelidae), followed by several Hymenoptera families such as Halictidae, Apidae and Megachilidae (Fig. 6 – right column). On the other hand, the species diversity during summer was largely dominated by Hymenoptera (~ 49%), with many species recorded from the Megachilidae, Apidae and Halictidae families. Coleoptera represented ~ 25% of the species diversity, the families Mordellidae, Buprestidae and Chrysomelidae being the most represented. On the other hand, the hoverflies showed a high level of diversity even though they were not abundant (41 species with 318 individuals). Similar seasonal insect communities have been identified in other studies using temporal surveys, particularly in Mediterranean climates, exhibiting largely the same pattern — except for one major difference in studies conducted at higher altitudes (Herrera 1988, Petanidou and Ellis 1993, Bosch et al. 1997, Herrera 2018, Morente-López et al. 2018, Aitakka et al. 2024). Anthophilous Coleoptera are mostly and strongly active in spring and summer in Mediterranean Region and their diversity decreases with (increasing) latitudes, while the greatest diversity of Hymenoptera can be found in Mediterranean environments (Herrera 1988, Moore et al. 2018, Ollerton 2021, Maestracci et al. 2024).

**Figure 7.**
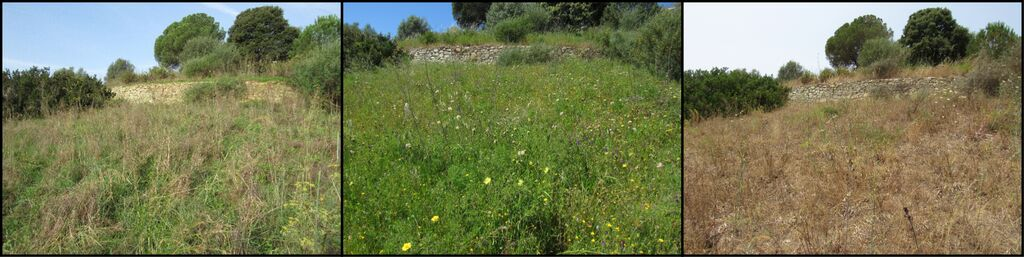
Floral composition in November, April and July for the Loretto site illustrating the habitat dynamism.

This seasonal structure of the anthophilous insect community reflects insect and plant annual phenologies, for example, Hymenoptera were active all year-round, beetles mostly in spring and summer and hoverflies mostly in autumn-winter. The flowering species changed also seasonally as illustrated on the site of Loretto (Fig. 7). In winter-autumn, three main angiosperm families were flowering: the Asteraceae (*Dittrichiaviscosa*, for example), the Apiaceae (*Foeniculumvulgare)* and the Brassicaceae (*Raphanus raphanistrum)*. In spring, the Asteraceae were the dominant flowering family totalling 41% of visits particularly on *Urospermumdalechampii*, other families being the Brassicaceae, the Apiaceae and the Cistaceae to a lesser extent. Finally in summer, the two main flowering plant families were the Apiaceae (*Daucuscarota* and *Foeniculumvulgare*) and the Asteraceae (*Carlinacorymbosa*).

### Conclusion and perspectives

In a previous paper (Maestracci et al. 2024), we highlighted that the abundance and diversity of the studied anthophilous insect communities were not linked and the same trend is observed here. Specifically, Coleoptera is the most abundant order, while Hymenoptera is the most diverse order amongst anthophilous insects. The community composition varied significantly throughout the year, with three distinct communities corresponding to the winter-autumn, spring and summer seasons, which is consistent with other studies in the Mediterranean Region (Herrera 1988, Petanidou and Ellis 1993). This supports the notion that, in environments with marked phenological patterns, such dynamism must be considered for a better understanding of ecosystem functioning.

We adopted a broader approach by considering the four main orders of anthophilous insects, rather than focusing solely on taxa that are currently well known (Requier et al. 2024). For example, Coleoptera are regularly mentioned as frequent and abundant floral visitors, but their species appeared to be often neglected and their role as pollen vectors may consequently be underestimated (Wardhaugh 2015, Rader et al. 2016, Sayers et al. 2019). However, this approach is time-consuming because it faces challenges related to family taxonomical revisions particularly for neglected taxa.

## Supplementary Material

48B435E3-95FD-53CD-A3DD-3B87938078A2 10.3897/BDJ.13.e144560.suppl1 Supplementary material 1 Anthophilous insects Data type Morphospecies and species Brief description List of morphospecies and species of insects of thermo-Mediterranean schrubland maquis (Ajaccio, Corsica, France) 2023. File: oo_1182828.txt https://binary.pensoft.net/file/1182828 Pierre-Yves Maestracci; Laurent Plume and Marc Gibernau

7215802E-9A92-5933-80D2-DF94D48AD5CD 10.3897/BDJ.13.e144560.suppl2 Supplementary material 2 Diversity indices with i-Chao1 Data type Diversity indices and richness estimator Brief description Diversity indices with i-Chao1 (estimator of total species richness taking account number of singletons, doubletons and species sampled 3 or 4 times), completeness (number of species / estimator * 100) and number of singletons per order for 2022, 2023 and 2022 + 2023. File: oo_1222135.csv https://binary.pensoft.net/file/1222135 Pierre-Yves Maestracci

DB8F051B-F545-5030-9203-5A062A07CA46 10.3897/BDJ.13.e144560.suppl3 Supplementary material 3 Family identities for graphs 3 and 5 Data type Family names according to abbreviation used in graphs Brief description Family identities according to graphs 3 and 5, where only the first three letters are used. File: oo_1220508.txt https://binary.pensoft.net/file/1220508 Pierre-Yves Maestracci

79557D78-152B-5273-86BC-57874DE97C98 10.3897/BDJ.13.e144560.suppl4 Supplementary material 4 One-way ANOSIM (Bonferroni-corrected p values) Data type Table associated with NMDS Brief description One-way ANOSIM (Bonferroni-corrected p values). File: oo_1228606.csv https://binary.pensoft.net/file/1228606 Pierre-Yves Maestracci and Marc Gibernau

## Figures and Tables

**Table 1.** Studied sites and detailed main characteristics (geographical and vegetation).

Locality	Geographical coordinates		Orientation	Main Vegetation	Area (ha)	Effective studied area (ha)
	Decimal latitude and longitude	Altitude (m)				
Loretto	41.933698, 8.718367	85	S	Wasteland [CORINE-Biotope: 87.1); Matorral with olive trees and mastic trees [CORINE-Biotope: 32.12)	1.9	0.33
Suartello	41.953102, 8.755813	90	SSE	Grassland [CORINE-Biotope: 34.4]; High maquis of the western Mediterranean [CORINE-Biotope: 32.311]	2.5	0.89
Vignola	41.912298, 8.650145	30	SW	Medium maquis with *Cytisuslaniger* and *Pistacialentiscus* in mosaic with *Oleaeuropea* – Fruity calicotome [CORINE-Biotope: 32.215]; Maquis with *Cistusmonspeliensis* [CORINE-Biotope: 32.341]	18	1.27
